# Scorpionfish rapidly change colour in response to their background

**DOI:** 10.1186/s12983-023-00488-x

**Published:** 2023-03-03

**Authors:** Leonie John, Matteo Santon, Nico K. Michiels

**Affiliations:** 1 Animal Evolutionary Ecology, Institute of Evolution and Ecology, University of Tübingen, Auf Der Morgenstelle 28, 72076 Tübingen, Germany; 2 Ecology of Vision Group, School of Biological Sciences, University of Bristol, 24 Tyndall Avenue, Bristol, BS8 1TQ UK

**Keywords:** Background matching, Calibrated image analysis, Camouflage, Colour change, Predator–prey interactions, Scorpionfish, Visual modelling, Biofluorescence

## Abstract

**Background:**

To facilitate background matching in heterogenous environments, some animals rapidly change body colouration. Marine predatory fishes might use this ability to hide from predators and prey. Here, we focus on scorpionfishes (Scorpaenidae), well-camouflaged, bottom-dwelling sit-and-wait predators. We tested whether *Scorpaena maderensis* and *Scorpaena porcus* adjust body luminance and hue in response to three artificial backgrounds and thereby achieve background matching. Both scorpionfish species are also red fluorescent, which could contribute to background matching at depth. Therefore, we tested whether red fluorescence is also regulated in response to different backgrounds. The darkest and the lightest backgrounds were grey, while the third background was orange of intermediate luminance. Scorpionfish were placed on all three backgrounds in a randomised repeated measures design. We documented changes in scorpionfish luminance and hue with image analysis and calculated contrast to the backgrounds. Changes were quantified from the visual perspective of two potential prey fishes, the triplefin *Tripterygion delaisi* and the goby *Pomatoschistus flavescens*. Additionally, we measured changes in the area of scorpionfish red fluorescence. Because scorpionfish changed quicker than initially expected, we measured luminance change at a higher temporal resolution in a second experiment.

**Results:**

Both scorpionfish species rapidly adjusted luminance and hue in response to a change of background. From prey visual perspective, scorpionfishes’ body achromatic and chromatic contrasts against the background were high, indicating imperfect background matching. Chromatic contrasts differed considerably between the two observer species, highlighting the importance of choosing natural observers with care when studying camouflage. Scorpionfish displayed larger areas of red fluorescence with increasing luminance of the background. With the second experiment, we showed that about 50% of the total luminance change observed after one minute is achieved very rapidly, in five to ten seconds.

**Conclusion:**

Both scorpionfish species change body luminance and hue in response to different backgrounds within seconds. While the achieved background matching was suboptimal for the artificial backgrounds, we propose that the observed changes were intended to reduce detectability, and are an essential strategy to camouflage in the natural environment.

**Supplementary Information:**

The online version contains supplementary material available at 10.1186/s12983-023-00488-x.

## Background

Background matching, where body colouration and pattern of an animal are similar to the background, is one of the most common strategies to hide from predators or prey [1, 2]. To match the background in a heterogenous environment, animals may have a fixed colour and pattern that performs sub-optimally with a wide range of backgrounds, actively choose matching substrates by relocating, or adjust their appearance in response to backgrounds by changing colour and pattern [3, 4]. Depending on the underlying mechanism, this colour change can happen rapidly, over seconds to a few minutes, or slowly, over hours or days or even months [3, 5, 6]. Rapid colour change is mediated by chromatophores containing pigment organelles that can be aggregated or dispersed within the cell [5]. Depending on the pigment, chromatophores can be divided into different types. While melanophores are the type that typically regulates luminance change, others allow changes in hue and/or saturation [5]. This physiological, rapid colour change for camouflage has been documented in reptiles [7, 8], and in marine animals such as cephalopods (e.g. [9, 10]). Only a few fish species have been studied in this context, for example flatfish [11, 12] and rock pool gobies [13–15]. Studies that empirically measure rapid colour change for camouflage from the visual perspective of natural observers are scarce [7, 13–15].

Red (long wavelength) fluorescence is a widespread component of body colouration in fishes, and is particularly common among gobies (*Bryaninops*, *Eviota*), triplefins (*Enneapterygius*, *Tripterygion*), dragonets (*Synchiropus*) and small wrasses (*Cirrhilabrus, Paracheilinus*), but also larger cryptic predatory fishes [16, 17]. With increasing depth in marine environments, longer wavelengths are absorbed faster than shorter wavelengths, resulting in a blue-green shifted light environment below ten meters [18]. Hence, red reflective objects appear dull grey at such depth, whereas red fluorescent structures can still show subtle grades of redness because they absorb short (blue-green) light and re-emit the energy at longer (red) wavelengths. Many marine substrates are red fluorescent, particularly when dominated by calcareous algae and other sedentary organisms such as corals [19]. For cryptic and benthic fishes, such as the scorpionfishes, it has therefore been suggested that red fluorescence contributes to background matching at depth as a subtle but possibly important colour component [16, 20].

The scorpionfishes (Scorpaenidae) are a family of benthic predators that rely on camouflage for hunting. As sit-and-wait predators, they remain motionless until prey comes close enough to be caught rapidly via suction feeding. Such ambush predators therefore face strong pressure to evolve particularly good camouflage [21]. Background matching can help to decrease detectability by prey [1] and could therefore increase foraging success. Colour change has the potential to allow for background matching on various substrates, generating a broader range of suitable microhabitats for hunting [21]. Given their wide distribution, high species diversity, benthic sit-and-wait predation tactic and diverse camouflage strategies, scorpionfish are an ideal system for experimental studies of fish camouflage. Yet, research on this topic is rare [22].

**Fig. 1:**
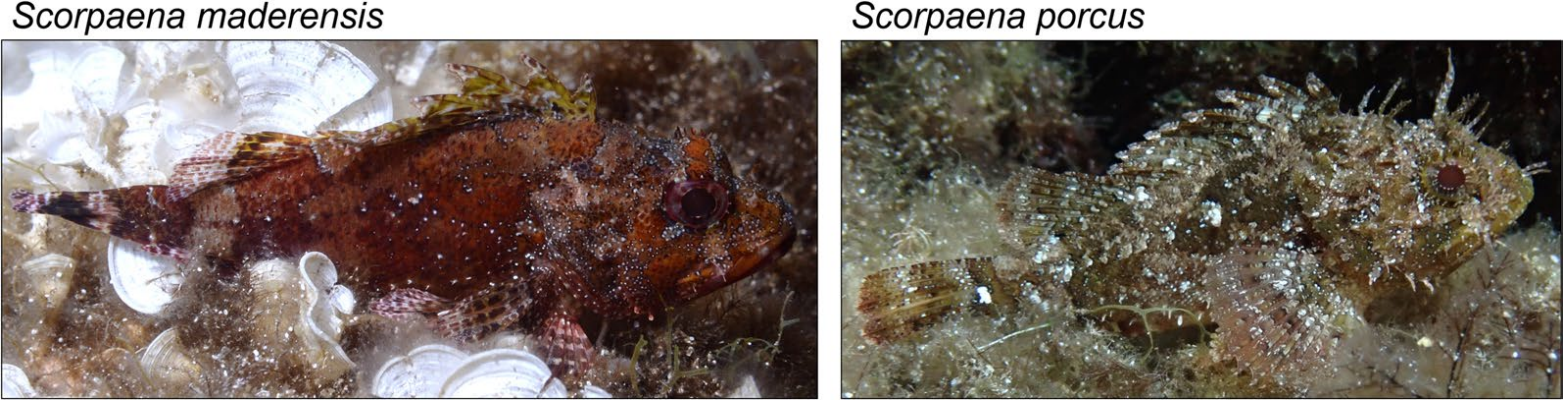
*Scorpaena maderensis* (left) and *S. porcus* (right) in their natural environment. Photos by LJ

In this study, we explored colour change in scorpionfishes. We chose to test two species, *Scorpaena maderensis* and *Scorpaena porcus* (Fig. 1), to understand whether colour change would be species-specific. We tested whether (1) scorpionfish rapidly change their body luminance and hue when placed on different backgrounds, and (2) how well they match their background by doing so. Such results may depend on the visual system of the observer, which is highly variable in marine animals [23, 24]. We therefore assessed the objectives from the visual perspective of two prey fish species as ecologically relevant observers with differing spectral sensitivity, the triplefin *Tripterygion delaisi* and the goby *Pomatoschistus flavescens*. To test objective (1), we placed individual scorpionfish of the two species on three artificial backgrounds: (a) low luminance, achromatic *dark/grey*, (b) medium luminance, chromatic *medium/orange*, and (c) high luminance, achromatic *light/grey*. We expected both scorpionfish species to change luminance and show the lowest luminance on the *dark/grey* background, medium luminance on the *medium/orange* background and highest luminance on the *light/grey* background. As for the hue, we expected scorpionfish to show a similar hue on the *dark/grey* and the *light/grey* backgrounds, but hue to be shifted to longer wavelengths on the *medium/orange* background. We quantified scorpionfish body luminance and hue based on cone catches for the two observers at one and five minutes after relocation to a new background. To test objective (2), we assessed the degree of background matching by calculating achromatic and chromatic contrast of scorpionfish body against the background from the visual perspective of the same two observers. We expected that scorpionfish display a similar luminance and hue to the background and therefore show a low contrast on all backgrounds. We expected both scorpionfish species to show a similar degree of background matching. We also tested whether (3) red fluorescence is part of the expected hue change mechanism. We therefore measured the total area of scorpionfish body showing red fluorescence when placed on the different backgrounds. We expected fish to show more fluorescence on the *medium/orange* background compared to the other backgrounds, analogous to the expected hue change. Because both scorpionfish species occur in shallow water but can also be found at depths of 30–40 m [25, 26], regulating red fluorescence together with red reflectance could enhance background matching at depths were long-wavelength light is scarce. In this first experiment, we observed that luminance and hue changes were happening faster than initially expected, i.e. in less than a minute. To (4) quantify how rapid this change was, we conducted a second experiment where we documented body luminance of scorpionfish every five seconds for 30 s, after relocation from a black to a white background.

## Results

### Changes in luminance and hue

**Fig. 2:**
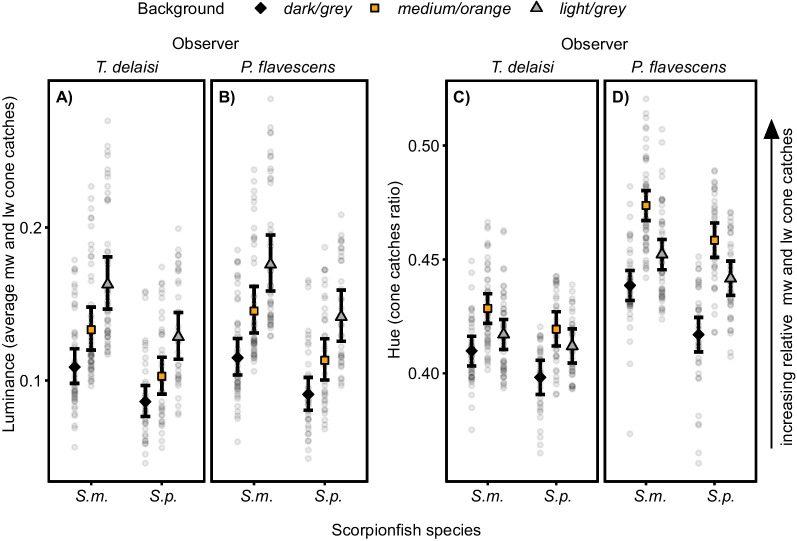
Scorpionfish luminance and hue change across backgrounds. Scorpionfish luminance (average of the medium (mw) and long wavelength (lw) cone catches) from **A** *Tripterygion delaisi* and **B** *Pomatoschistus flavescens* visual perspective. Scorpionfish hue (ratio of short compared to medium and long wavelength cone catches, where higher values indicate a shift towards longer wavelengths, see Methods) from **C** *T. delaisi* and **D** *P. flavescens* visual perspective. *S. m.* = *Scorpaena maderensis*, *S. p.* = *S. porcus*. All panels show model estimates and raw data for all combinations of *background*, *species* and *observer*. Each point represents a measurement for each individual fish (N = 24 *S. maderensis*, N = 18 *S. porcus*) averaged over the two time points (after one and five minutes adaptation time to the backgrounds, see Methods). Markers with vertical bars represent predicted mean and 95% compatibility intervals (CIs) derived from 10,000 simulations of the posterior distribution of model parameters. The strength of the difference between two groups increases with decreasing degree of overlap of their 95% CIs

Both scorpionfish species changed luminance according to the background (Fig. 2A, B). Scorpionfish body luminance differed for all background comparisons, for both scorpionfish species and regardless of observer (see Table 1A, median differences and 95% CIs deviate from zero for any given comparison). As expected, mean luminance of both scorpionfishes was lowest on the *dark/grey* background, intermediate on the *medium/orange*, and highest on the *light/grey* background (Fig. 2A, B, Table 1A), showing that the observed body luminance change follows the direction of luminance change of the background. Luminance of *Scorpaena maderensis* was overall higher than that of *S. porcus* (median difference of luminance averaged over *background* and *observer*: 0.029, 95% CI 0.011 to 0.049). Comparing the two observers, results for luminance change were similar (Table 1B, compare median differences for the same scorpionfish species and background comparison between the section “Observer = *T. delaisi*” and section “Observer = *P. flavescens*”, 95% CIs overlap). Scorpionfish had on average a slightly higher luminance from *P. flavescens* visual perspective (Fig. 2B) than from *T. delaisi* visual perspective (Fig. 2A) (median difference of scorpionfish luminance averaged over *background* and *species:* 0.010, 95% CI 0.008 to 0.012).

**Table 1:** Median differences in luminance and hue between all background combinations

	*Scorpaena maderensis*			*Scorpaena porcus*		
Background	Median	Lower CIs	Upper CIs	Median	Lower CIs	Upper CIs
**(A) Luminance.** R^2^_marg_ = 0.387, R^2^_cond_ = 0.937						
Observer = *T. delaisi*						
Medium/orange–dark/grey	0.024	0.020	0.030	0.017	0.013	0.021
Medium/orange–light/grey	− 0.030	− 0.036	− 0.024	− 0.026	− 0.032	− 0.021
Light/grey–dark/grey	0.054	0.047	0.062	0.042	0.036	0.049
Observer = *P. flavescens*						
Medium/orange–dark/grey	0.030	0.025	0.036	0.022	0.018	0.027
Medium/orange–light/grey	− 0.030	− 0.037	− 0.025	− 0.028	− 0.034	-0.023
Light/grey–dark/grey	0.061	0.054	0.069	0.051	0.044	0.059
**(B) Hue.** R^2^_marg_ = 0.597, R^2^_cond_ = 0.881						
Observer = *T. delaisi*						
Medium/orange–dark/grey	0.019	0.015	0.023	0.021	0.017	0.026
Medium/orange–light/grey	0.011	0.008	0.015	0.007	0.003	0.012
Light/grey–dark/grey	0.007	0.003	0.011	0.014	0.009	0.018
Observer = *P. flavescens*						
Medium/orange–dark/grey	0.035	0.031	0.039	0.042	0.037	0.046
Medium/orange–light/grey	0.022	0.018	0.025	0.017	0.012	0.021
Light/grey–dark/grey	0.014	0.010	0.017	0.025	0.020	0.029

Median differences of A) luminance and B) hue between all combinations of *background*, *species* and *observer*. Estimated effect sizes are reported as the median difference and its 95% compatibility intervals (CIs), calculated from 10,000 simulations of the posterior distribution of model parameters. N = 24 for *S. maderensis* and N = 18 for *S. porcus*. Effect size strength increases with increasing deviation of median differences from zero, and the robustness of the result increases with decreasing degree of overlap of the 95% compatibility intervals (CIs) with zero

Both scorpionfishes also changed hue in response to the background (Fig. 2C, D). Scorpionfish body hue differed for all background comparisons for both scorpionfishes and regardless of the observer (see Table 1B, median differences and 95% CIs deviate from zero for any given comparison). As expected, mean hue was shifted towards longer wavelengths (i.e. a higher hue value) for both scorpionfishes on the *medium/orange* background compared to the *dark/grey* and *light/grey* background (Fig. 2C, D, Table 1B). Hue also differed on the *light/grey* and *dark/grey* backgrounds (Table 1B, see *light/grey*–*dark/ grey* comparisons), being shifted towards longer wavelengths on the *light/grey* background (Fig. 2C, D). In general, hue of *S. maderensis* was more long-wavelength shifted compared to *S. porcus* (median difference of hue averaged over *background* and *observer*: 0.012, 95% CI 0.003 to 0.021). Hue perception was different depending on the observer, hue changes were stronger from *P. flavescens* compared to *T. delaisi* visual perspective (Table 1B, compare median differences for the same scorpionfish species and background comparison between the section “Observer = *T. delaisi*” and section “Observer = *P. flavescens*”, 95% CIs mostly do not overlap). Scorpionfish had on average a more long-wavelength shifted hue from *P. flavescens* (Fig. 2D) compared to *T. delaisi* visual perspective (Fig. 2C) (median difference of scorpionfish hue averaged over *background* and *species:* 0.033, 95% CI 0.032 to 0.035).

### Background matching

**Fig. 3:**
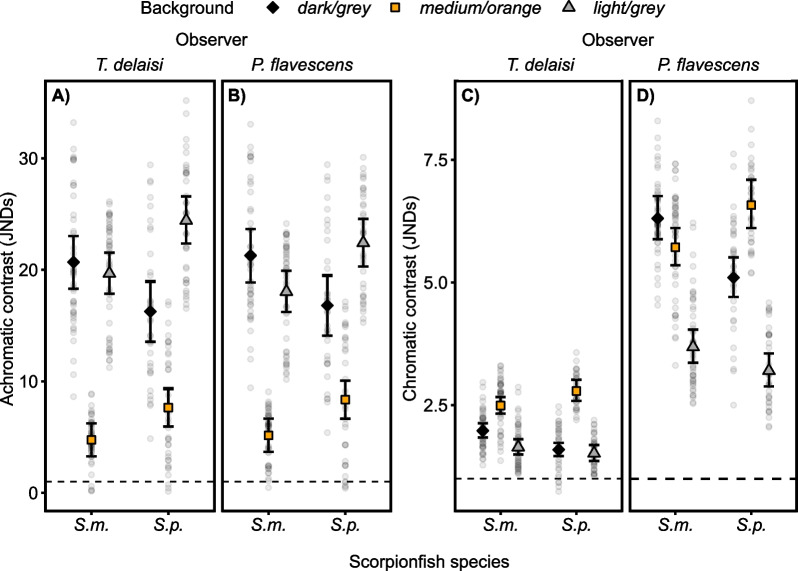
Achromatic and chromatic contrasts of scorpionfish body against the background are above detection threshold. Achromatic contrast from **A** *Tripterygion delaisi* and **B** *Pomatoschistus flavescens* visual perspective in Just Noticeable Differences (JNDs). Chromatic contrasts from **C** *T. delaisi* and **D** *P. flavescens* visual perspective in JNDs. Dashed line = detection threshold of one JND. *S. m.* = *Scorpaena maderensis*, *S. p.* = *S. porcus*. All panels show model estimates and raw data for all combinations of *background*, *species* and *observer*. Each point represents a measurement for each individual fish (N = 24 *S. maderensis*, N = 18 *S. porcus*) averaged over the two time points (after one and five minutes adaptation time to the backgrounds, see Methods). Markers with vertical bars represent predicted mean and 95% compatibility intervals (CIs) derived from 10,000 simulations of the posterior distribution of model parameters. The strength of the difference between two groups increases with decreasing degree of overlap of their 95% CIs

Mean achromatic contrast of scorpionfish body against the background was above the detection threshold on all backgrounds, regardless of scorpionfish or observer species (Fig. 3A, B, all predicted means and their 95% CIs are above one JND). Both scorpionfish species showed the lowest mean achromatic contrast on the *medium/orange* background (Fig. 3A, B, 95% CIs of predicted means do not overlap with *dark/grey* or *light/grey*). Achromatic contrast was similar from both visual perspectives (Fig. 3A, B) (median difference of scorpionfish body achromatic contrast against the background averaged over *background* and *species:* 0.23, 95% CI  − 0.04 to 0.49).

Mean chromatic contrast of scorpionfish body against the background was above detection threshold on all backgrounds, regardless of scorpionfish or observer species (Fig. 3C, D, all predicted means and their 95% CIs are above one JND). On which background scorpionfish had the lowest and highest mean chromatic contrast was depending on scorpionfish and observer species (Fig. 3C, D, see Additional file 1: Table S1B for all comparisons between chromatic contrast on all backgrounds). Chromatic contrast was clearly higher when calculated from *P. flavescens* visual perspective (Fig. 3D) compared to *T. delaisi* visual perspective (Fig. 3C) (median difference of scorpionfish body chromatic contrast against the background averaged over *background* and *species*: 3.11, 95% CI 3.03 to 3.20).

### Changes in fluorescence

**Fig. 4:**
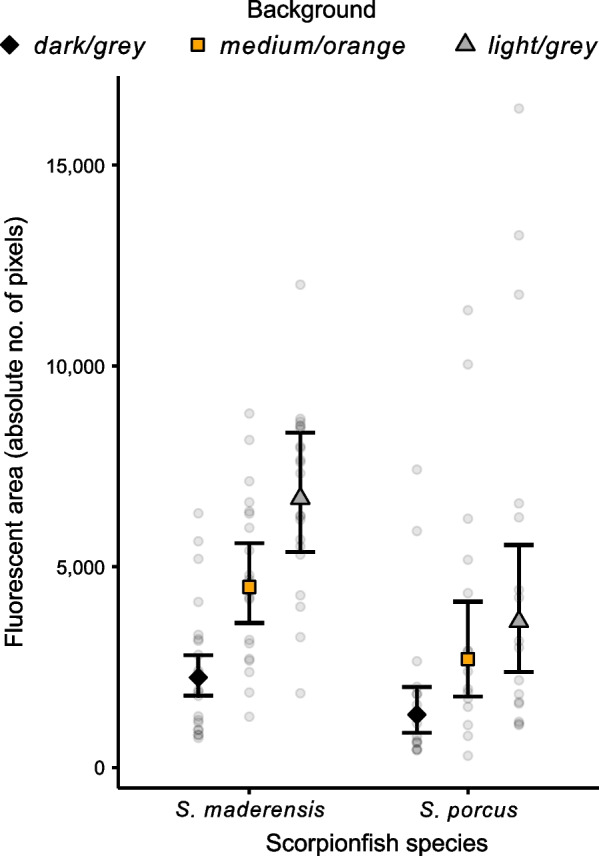
Fluorescent area of scorpionfish body increases with background luminance. The figure shows model estimates and raw data for each background and scorpionfish species. Each point represents a measurement for each individual fish (N = 21 *Scorpaena maderensis*, N = 16 *S. porcus*). Fluorescent area is given in absolute pixel count. Markers with vertical bars represent predicted mean and 95% compatibility intervals (CIs) derived from 10,000 simulations of the posterior distribution of model parameters. The strength of the difference between two groups increases with decreasing degree of overlap of their 95% CIs

The fluorescent area varied between all backgrounds for both scorpionfish species (see Table 2, median differences and CIs deviate from zero for any given comparison). Against our expectations, mean fluorescent area was not largest on the *medium/orange* background, but increased with increasing background luminance in both species (Fig. 4, Table 2, see *medium/orange*–*dark/grey* and *medium/orange–light/grey* comparisons, median differences and CIs deviate from zero, where fluorescent area is larger on the *light/grey* than on the *medium/orange* background). Across all backgrounds, *S. maderensis* showed a larger fluorescent area than *S. porcus* (median difference of *fluorescent area* between *species*, averaged over *background*: 1930.71 pixels, 95% CI 533.76 to 3137.43).

**Table 2:** Median differences in fluorescent area of scorpionfish body across backgrounds for both scorpionfish species

	*Scorpaena maderensis*			*Scorpaena porcus*		
Background	Median	Lower CIs	Upper CIs	Median	Lower CIs	Upper CIs
Difference in fluorescent area (absolute pixel count). R^2^_marg_ = 0.373, R^2^_cond_ = 0.804						
Medium/orange–dark/grey	2244.95	1399.03	3278.23	1364.06	737.62	2350.44
Medium/orange–light/grey	− 2213.41	− 3711.56	− 897.32	− 924.62	− 2048.1	− 55.90
Light/grey–dark/grey	4457.48	3191.07	5957.11	2307.90	1357.15	3674.72

Estimated effect sizes are reported as the median difference and its 95% compatibility intervals (CIs), calculated from 10,000 simulations of the posterior distribution of model parameters. N = 21 for *Scorpaena maderensis* and N = 16 for *S. porcus*. Effect size strength increases with increasing deviation of median differences from zero, and the robustness of the result increases with decreasing degree of overlap of the 95% compatibility intervals (CIs) with zero

### Rate of luminance change

**Fig. 5:**
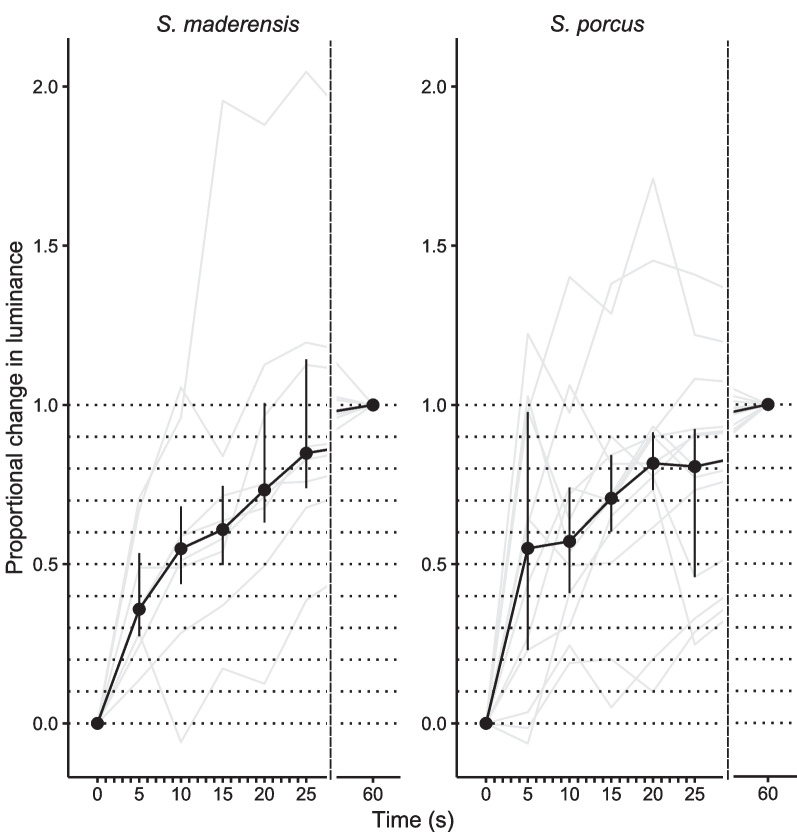
Time needed for *Scorpaena maderensis* and *S. porcus* to change body luminance. Figure shows median (points) and interquartile range (vertical bars) of the proportional change in body luminance every five seconds for 25 s, relative to initial (*y* = 0) and final luminance (*y* = 1) measured for each individual (see Methods). The black curve connects medians for each time point. The grey lines connect each data point per individual fish. The dotted horizontal lines indicate every 10% step from 0 to 100%, the dashed vertical line represents is a gap with no data between 25 and 60 s. N = 9 for *S. maderensis* and N = 13 for *S. porcus*

*S. maderensis* individuals took on average about 10 s to achieve 50% and 23 s to achieve 80% of the body luminance change measured over the observation time of 60 s (Fig. 5). For *S. porcus*, more than 50% of the change was already achieved after 5 s, and 80% after 20 s (Fig. 5).

## Discussion

### Changes in luminance and hue

As expected, both species of scorpionfish changed their body luminance according to the luminance of the background. The lowest body luminance was observed on the *dark/grey* background, intermediate luminance on the *medium/orange* and the highest luminance on the *light/grey.* Scorpionfish also changed their body hue to longer wavelengths when placed on the *medium/orange* background compared to the other two backgrounds. Luminance and hue change were also connected, as shown by the shift in hue between the *dark/grey* and the *light/grey* background. The two grey backgrounds had a chromatic contrast below detection threshold from scorpionfish visual perspective (see Methods), and we therefore expected scorpionfish to display a similar hue on both backgrounds. However, in natural environments, changes of background luminance and hue usually come together, especially for carotenoid-based colours [27]. This dependence of luminance and hue occurrence and perception might explain the observed shift in scorpionfish body hue on the lighter background. Another reason for the observed shift in hue between the grey backgrounds could be a passive hue change as the scorpionfish changed luminance. Although the proximate mechanisms of colour change have not been investigated in scorpionfish, the observed colour change is probably due to the aggregation or dispersion of pigment organelles in chromatophores, a common mechanism present in many fish species [28]. Luminance changes are probably mediated by melanophores [5], and pigment organelle aggregation in the melanophores might have affected the hue of the scorpionfish as well, e.g. by exposing underlying structures in the fish skin [29]. However, the stronger change in body hue towards longer wavelengths on the *medium/orange* background compared to both grey backgrounds indicates that hue can be regulated actively, by an additional type of chromatophores. If luminance and hue change were mediated only by the same type of chromatophores, the long wavelength shift in body hue should have shown a similar pattern to luminance change across all three backgrounds. Such fine regulation of luminance and hue by different chromatophore types could allow scorpionfish to camouflage on different backgrounds [5, 13]. Both scorpionfish species tested showed similar results, suggesting that such colour change mechanisms may be present across the family Scorpaenidae, which are all benthic ambush predators. Still, *S. maderensis* appeared redder and lighter on each background compared to *S. porcus*. These species-specific differences might be related to differences in the species’ ecology, or to different camouflage strategies [30]. Possible defining factors, e.g. microhabitat use and related background preferences, are however unknown for these two species or any other scorpionfish. Our study shows to what extent these species can adjust body luminance and hue, which is valuable information for further studies investigating their camouflage on natural backgrounds.

### Background matching

Contrary to our expectations, scorpionfish did not match the artificial backgrounds very well. Achromatic contrasts of fish body against the backgrounds were clearly above detection threshold, especially on the two grey backgrounds. Yet, the fish did show a strong luminance change in the predicted direction, which likely reduced the contrast to background luminance. Moreover, on backgrounds that are difficult to match, fish may rely on other camouflage strategies such as disruptive colouration [31], which we did not quantify here. It is possible that fish changed colour to increase disruption, e.g. by changing certain patches in their pattern to increase pattern contrast or facilitate differential blending [32]. The poor achromatic match we observed may be explained by our use of artificial backgrounds of extremely low and high luminance, which might differ to the luminance range of natural backgrounds. Similarly, the orange hue we used might have been too artificial for the scorpionfishes, which may explain that even though fish adjusted body hue towards longer wavelengths on the *medium/orange* background, they still had high contrast to this background. Another explanation for this could be the scorpionfish’s limited ability to discriminate long wavelengths given their spectral sensitivity [33]. We cannot exclude that longer adaptation time would have allowed for further improvement of background matching [7, 34, 35]. Even though neither scorpionfish species matched the backgrounds well when considering both achromatic and chromatic contrast, the response into the predicted directions suggests the luminance and hue changes were meant to improve background matching. Further studies are needed to test how well scorpionfish can match the background of natural substrates and which further camouflage strategies are deployed.

While changes in body luminance and achromatic contrasts against the backgrounds were comparable for both modelled observers, this was different for body hue and chromatic contrasts. Chromatic contrast of scorpionfish body against the backgrounds was higher from *P. flavescens* than from *T. delaisi* visual perspective, where it was below three JNDs on all backgrounds. While we do not have behavioural data on actual detection thresholds in *T. delaisi*, a conservative approach of three JNDs as detection threshold has been used for many animals including fishes [13, 35, 36], indicating that the chromatic contrast would be difficult to perceive at least from *T. delaisi* visual perspective. *P. flavescens* is a trichromat with a spectral sensitivity shifted to longer wavelengths compared to *T. delaisi*, which explains the better colour discrimination in the long wavelengths. These results highlight the importance to consider different observers when investigating animal colour change. In cases where scorpionfish match background luminance well, chromatic contrast might still reveal them to certain observers.

### Changes in fluorescence

We predicted that red fluorescence would be upregulated on the *medium/orange* background. Even though our experiment was carried out under surface light conditions, we expected fluorescence to be increased on a red reflective background, since we did not expect the fish to have a physiological colour change mechanism that would be regulated differently depending on a specific light environment. Contrary to our prediction, the area of scorpionfish body showing fluorescence was not largest on the orange background, but on the lightest background. This suggests that display of red fluorescence depends on background luminance. This may be a consequence of melanosome aggregation on light backgrounds, an effect also known from other fishes [29]. How strong the contribution of red fluorescence is relative to reflectance in this experiment, or at depth, where red reflectance is much lower [16, 20], cannot be assessed with our data.

### Rate of luminance change

Comparing the measurements taken after one and five minutes, luminance did not change much anymore, indicating that changes took place within one minute, before the first photo in experiment 1 was taken. A separate assessment of the rate of luminance change in experiment 2 showed that about 80% of the change achieved after one minute happened already within the first 20 to 25 s. More than 50% of the change was achieved after 10 s in *Scorpaena maderensis*, but already after 5 s in *S. porcus*. Such rapid colour change for camouflage is also known from tropical flounders [11].

## Conclusions

This is the first study investigating whether scorpionfish adjust body luminance and hue to a given background. While fish were unable to match the extreme, artificial backgrounds below detection threshold, we show that both species rapidly change colour in the expected direction. As sit-and-wait predators, scorpionfish are an ideal group to study camouflage of predators from prey visual perspective. While this study focussed on two species of scorpionfish and tested background matching only, there are more species and types of camouflage worth exploring in this family.

## Methods

### Study species

The first experiment was carried out in the Station de Recherches Sous-marines et Océanographiques (STARESO), Corsica, France in June and July 2021. The second experiment was carried out in the same location in July 2022. Madeira rockfish *Scorpaena maderensis* and the black scorpionfish *Scorpaena porcus* (Fig. 1) were caught with hand nets while SCUBA diving under the station’s general sampling permit. All fish were kept in flow-through tanks (125 × 55 × 58 cm/400 L). Both species are ambush predators that sit motionless between rocks or algae and sedentary animals on natural hard substrates [25]. Scorpionfish are generalists that feed on a variety of small fishes and invertebrates. Both species mainly occur above 30–40 m [25, 26]. Fish sampled for our study were collected in 2–10 m depth. Observations under natural light conditions in the field indicate that both species can change colour, and that they are red fluorescent (personal observations).

### Experiment 1

#### Experimental setup

**Fig. 6:**
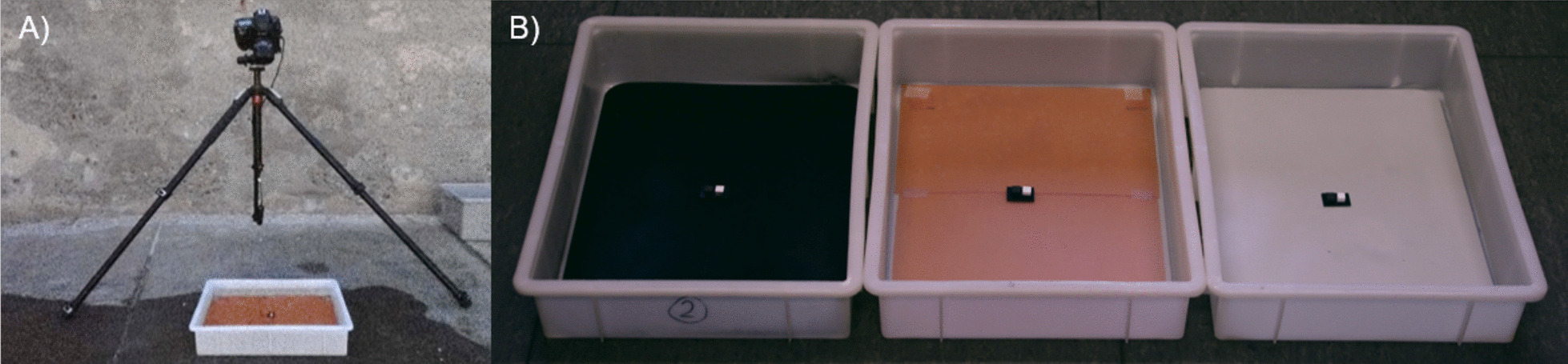
Setup of experiment 1. **A** Overview of the setup, **B** trays with the three backgrounds used in the first experiment (from left to right: *dark/grey*, *medium/orange*, *light/grey*)

To elicit changes in body colouration, fish were alternately placed in three white polyethylene trays (40 × 30 × 9 cm), each with a different uniformly coloured bottom (Fig. 6B). The walls of all trays were kept white. The three backgrounds were an achromatic, low luminance background (*dark/grey*), a chromatic, medium luminance background (*medium/orange*), and an achromatic, high luminance background (*light/grey*). We expected fish to show changes in luminance across all three backgrounds. Changes in hue on the orange background, but not on the grey backgrounds, would instead show that scorpionfish adjust body hue independently of luminance (see expectations in Introduction). We chose an orange reflective background to elicit hue changes in long wavelength body reflectance and fluorescence. If red fluorescence is part of dynamic background matching on long wavelength backgrounds, we expected to see a modulation of red fluorescence on the *medium/orange* background only. We did not test fish on fluorescent backgrounds or under deep-water light conditions since we did not expect the fish to distinguish between red fluorescence or reflectance, nor to have a physiological colour change mechanism that depends on the current light environment. We expected fish to simply regulate red fluorescence depending on the amount of red in the background, regardless of its origin. The *dark/grey* and *light/grey* backgrounds were plastic sheets spray-painted with black or light-grey spray paint (black: Marabou do it Colourspray black satin matt, Germany; light-grey: Maison Déco Relook Tout galet satin matt, France), and glued onto the bottom of the trays. The *medium/orange* background consisted of filter paper (LEE filter no. 204, Full C.T. Orange, Hampshire, UK) placed on the white bottom of the tray, and covered by a transparent plastic sheet. We chose to use filter paper for this background because all commercial orange spray paints we tried were fluorescent, which interfered with fish fluorescence photography (see below).

We quantified background appearance using a spectroradiometer (SpectraScan PR-740, Photo Research, New York, USA, with MS-75 standard lens) positioned on a tripod looking down at a 20° angle at the tray from a distance of ~ 100 cm to measure background reflectance relative to a diffuse white reflectance standard measured in the same way (SRS-99–010, Labsphere, NH, USA) (reflectance spectra in Additional file 1: Figure S2). To assess how scorpionfish would perceive the backgrounds, we calculated achromatic and chromatic contrasts between the backgrounds from a scorpionfish visual perspective by implementing their spectral sensitivities and cone ratio in the Receptor Noise Limited model [37] using the pavo R-package [38] in R (version 4.1.1) [39] (Table 3). *S. porcus* vision is characterized by short-wavelength single cones with average sensitivity peaking at 455 nm and medium-wavelength double cones with average sensitivity peaking at 530 nm ([33], as cited in [24]). The single to double cone ratio is 1:1 [40]. We assume similar visual properties for *S. maderensis*, for which there is no published record.

**Table 3:** Achromatic and chromatic contrasts between backgrounds from scorpionfish visual perspective

Backgrounds compared	Achromatic contrast (JND)	Chromatic contrast (JND)
Light/grey–dark/grey	20.95	0.99
Medium/orange–light/grey	9.29	5.38
Medium/orange–dark/grey	11.67	6.37

Contrasts are expressed in Just Noticeable Differences (JNDs) for each background comparison, as perceived by scorpionfish. Contrasts below one JND are not distinguishable, and increasing values indicate an increasing probability of detection [36]. All backgrounds differ in luminance (achromatic contrast). The medium luminance background (*medium/orange*) has a comparable achromatic distance to both the high and the low luminance background. Difference in colour (chromatic contrast) between the two grey backgrounds is not distinguishable, whereas the *medium/orange* background shows contrasts above detection threshold to both grey backgrounds

Each tray contained two centrally placed PTFE diffuse grey standards (12% and 72% grey, Berghof Fluoroplastic Technology GmbH, Eningen unter Achalm, Germany) and a scale bar (Fig. 6B). Trays were filled with fresh sea water before each trial. Trials took place outside in a shaded area under the open blue sky. Photos to document change in luminance and hue were taken with a calibrated Nikon D4 DLSR camera (NIKON CORPORATION, Tokyo, Japan, Micro-Nikkor 60 mm lens, RAW format, ISO and aperture fixed) positioned in the same way as the spectroradiometer (Fig. 6A).

Since reflectance and fluorescence both contribute to body colouration under daylight, we estimated changes in red fluorescence separately by using a 3D-printed, cylindrical photo-chamber that was placed over the scorpionfish on its current background (Additional file 1: Figure S3). The top-lid of the chamber included a ring-light source and camera-holder for an Olympus Tough TG-6 (Olympus Europa SE & Co. KG, Hamburg, Germany, RAW format, ISO and aperture fixed). The ring-light (WEEFINE ringlight 3000, WEEFINE Technology, China) was set to “blue” and covered with an additional cyan filter (LEE filter no. 172, Lagoon Blue, Hampshire, UK) to block wavelengths above 540 nm. The camera was instead equipped with a double red filter (LEE filter no. 106, Primary Red, Hampshire, UK) to block light below 580 nm. This combination of light and filters assured that only cyan excitation light reached the fish, and that only red fluorescent emission reached the camera.

#### Experimental procedure

**Fig. 7:**
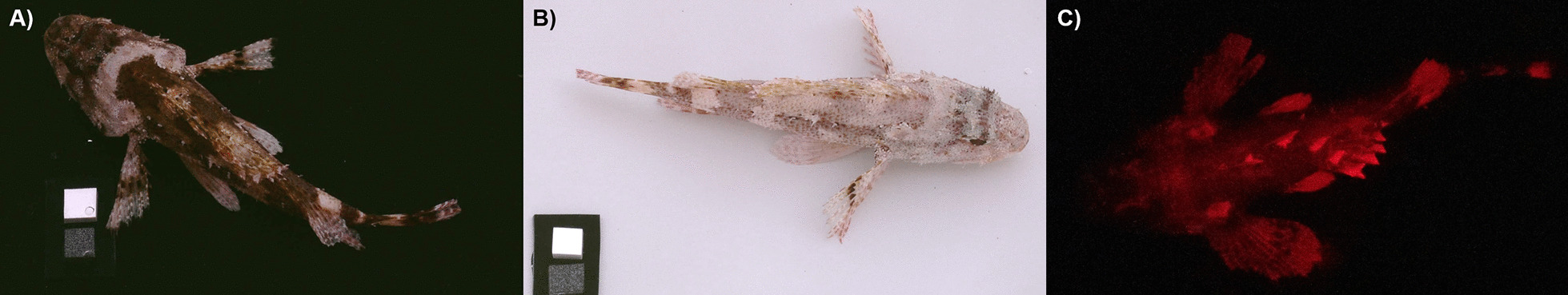
Scorpionfish can adjust body luminance, and display red fluorescence. Exemplary photos of the same *S. maderensis* individual **A** on the *dark/grey* and **B** on the *light/grey* background and **C** of a fluorescence photo of a different *S. maderensis* individual (adapted to the *dark/grey* background)

We tested 24 *S. maderensis* and 18 *S. porcus*. Mean standard length of both species was similar on average (*S. maderensis*: 7.04 ± 1.03 cm (mean ± SD), *S. porcus*: 7.03 ± 1.84 cm), and *S. porcus* had a slightly larger body area than *S. maderensis* on average when photographed from the top (*S. maderensis*: 7.58 ± 2.09 cm^2^, *S. porcus*: 8.41 ± 4.14 cm^2^). Each individual was tested on each background. At the start of the experiment, a fish was transferred into a medium luminance grey acclimation-box filled with fresh sea water, where it stayed for ten minutes. This acclimation period ensured initial short-term adaptation of each fish to the same background. Each fish was subsequently placed on the first of the three experimental backgrounds. It was photographed as soon as it settled (within a minute). A second photo was taken after five minutes adaptation time (Fig. 7A, B). Immediately after this, we placed the cylindrical photo-chamber on the fish, added a non-fluorescent red diffuse reflectance standard (SCS-RD-010, Labsphere, NH, USA) next to it, closed the chamber (details above), turned on the light source and took a photo (Fig. 7C). Taking a fluorescence photo took about 30 s. Subsequently, the fish was placed in the next tray and the procedure was repeated for the other two backgrounds. Exposing a fish to all backgrounds required around 20 min. The acclimation period was not repeated between backgrounds. In which order the fish were exposed to the three backgrounds was balanced across all individuals of a species to account for a potential effect of background order. After a completed trial, fish were either immediately brought back to the field or returned to a temporary housing tank. Each individual was used only once.

#### Image analysis

To quantify changes of luminance and hue between backgrounds, we used the Multispectral Image Analysis and Calibration (MICA) Toolbox plugin [41] for ImageJ (version 1.53o) [42]. Images were normalized with the 12% and 72% grey standards present in each tray, and converted into 32-bit multispectral images. For every image, we selected two regions of interests (ROI): (a) the ‘body’ of the fish, excluding the fins since they were transparent, and (b) a ca. 1 cm^2^ sample of the ‘background’ (for more detail on the ROI selection, see Additional file 1: Figure S4). We also measured standard length of each fish relative to the size standard and extracted the area of the fish body in cm^2^. All images were then batch-processed using a custom-written routine for MICA in ImageJ. First, reflectance images were converted to a cone-catch model, which included the spectral sensitivity of the camera and a modelled observer, and the spectra of photography and model illuminant, which were both a D65 spectrum. We chose D65 as the model illuminant since this was the light spectrum under which the experiment was run and under which the scorpionfish adjusted to the backgrounds. We modelled the vision of the yellow black-faced blenny *Tripterygion delaisi*, a common species and prey of scorpionfish. *T. delaisi* has single cones with average peak sensitivity at 468 nm, and double cones with average sensitivity peaking at 517 and 530 nm [43]. Since we were focusing on hue change in the long-wavelength part of the visible spectrum, we also modelled a natural observer with a better ability to perceive long wavelength changes, the two-spotted goby *Pomatoschistus flavescens*, which also occurs in the natural range of the scorpionfish. This fish has single and double cones with peak sensitivity at 456, 531 and 553 nm [44]. We assumed a Weber fraction of 0.05 for the most abundant cones and for the luminance channel for both species [45, 46], and a cone ratio (from shortest to longest wavelength photoreceptor) of 0.25:1:1 for *T. delaisi* [47] and 0.72:1:0.6 for *P. flavescens* [44, 48]. We defined the luminance channel as the average cone catches of the two longer wavelength sensitive cones, as fish likely perceive achromatic (luminance) contrasts through these photoreceptors [49]. The routine further processed the images to adjust for *T. delaisi* foveal spatial acuity of 7 cycles per degree [47] and 2.36 cycles per degree for goby vision [50] for a viewing distance of 30 cm by using the Gaussian Acuity Control and the Receptor Noise Limited (RNL) Ranked Filter functions of the MICA toolbox [51]. We then measured cone catches for the ROI ‘body’ and ‘background’ for both observers. To assess scorpionfish changes in luminance, we compared luminance channel cone catches measured for ‘body’ [35]. To assess changes in hue, we instead calculated the ratio of the difference between the cone catches of the short wavelength receptor and the sum of the two longer wavelength sensitive receptors and the total cone catches (*T. delaisi*: hue = ((λ_max_530 + λ_max_517) − λ_max_468) / (λ_max_530 + λ_max_517 + λ_max_468), *P. flavescens*: hue = ((λ_max_553 + λ_max_531) − λ_max_456) / (λ_max_553 + λ_max_531 + λ_max_456)), following previous studies [13, 14]. Finally, we calculated the contrast of fish against the background as perceived by the observers, to see how well scorpionfish were matching the backgrounds by comparing the ROI ‘body’ to the ROI ‘background’ for each image. Achromatic and chromatic contrasts were calculated implementing the Receptor Noise Limited model [37] informed with the cone catches of the three chromatic channels, and the luminance channel cone catches using the pavo R-package [38] in R, where we set weber fraction and cone ratios for each observer as described above [13, 14, 35, 36]. Contrasts are reported as Just Noticeable Differences (JNDs), where values below one JND indicate an indistinguishable contrast and higher values indicate an increased probability of detection [36, 37, 46].

Fluorescence photos were corrected for differences in shutter speed by adjusting exposure to the same speed for each photo of one individual in the program Olympus Workspace (version 1.5, OM Digital Solutions Corporation), and subsequently exported as TIF. Images were imported in ImageJ, and only the red channel was selected. To filter out noise, we removed all pixels with a brightness threshold below 100 (RGB scale), which was defined beforehand by manually testing different thresholds and identifying the most conservative threshold where background pixels (i.e. noise) were removed, but not pixels of the fish for any given background used. We counted the remaining pixels with ‘Analyse Particles’ to quantify changes fluorescent area within the fish body.

### Experiment 2

#### Experimental setup

To measure the rate of luminance change more precisely, we tested fish in a different setup. A white shallow plastic tray (40 × 60 × 9 cm) was divided into two compartments (40 × 30 × 9 cm each) by a removeable plastic wall. One compartment was kept white, while the other side was covered in black plastic. We chose to use black and white backgrounds instead of the same backgrounds as in experiment 1 since we wanted to record the fastest possible luminance change and we expected fish to change most rapidly if they would be moved between extremes. A moveable transparent plastic cylinder of 15 cm diameter and 8 cm height was placed in the tray. It had a small plastic edge at the bottom (2 × 1 cm) which served as a scale bar and on which two PTFE diffuse grey standards (12% and 72% grey, Berghof Fluoroplastic Technology GmbH, Eningen unter Achalm, Germany) were attached. To move the cylinder from the outside, it had a transparent handle reaching out of the tray. A Nikon D4 DLSR camera (NIKON CORPORATION, Tokyo, Japan, Micro-Nikkor 60 mm lens, RAW format, ISO and aperture fixed) was positioned on a tripod looking down at a 10° angle at the tray from a distance of ~ 120 cm.

#### Experimental procedure

To quantify the rate of luminance change, we tested 9 *S. maderensis* and 14 *S. porcus* in the setup for experiment 2. An individual was placed in the cylinder in the black compartment of the tray for one minute acclimation time. Then, the separating wall was pulled out and the fish was moved into the white compartment. We then took a photo every second for 30 s, and a last photo after 60 s. We assumed that the final luminance for short-term adaptation was achieved after this one minute since we observed in the first experiment that fish changed very little between one minute and five minutes adaptation time (Additional file 1: Figure S5). Fish were returned back into the field after the experiment.

#### Image analysis

Photos taken to measure the rate of luminance change were analysed with the same MICA toolbox routine used for experiment 1. We selected photos of the fish when first settled on the new background (second 0), and from second 5, 10, 15, 20, 25 and 60. For each fish, we only selected and measured a specific patch (Fig. 5A, dark dorsal patch behind the head framed by the gill covers), because this patch was easy to locate and select as an ROI in every individual regardless of its position. We then converted the images to *T. delaisi* vision as described above, and extracted luminance channel cone catches to test hypothesis 5). We chose to only present the data from *T. delaisi* vision as luminance perception of both observers is comparable (see Fig. 1A, B).

### Statistical analysis

#### Experiment 1

We implemented generalized linear mixed models with the glmmTMB R-package [52] following a custom-written guided linear modelling R-routine [53]. Model assessment followed the guidance of Santon et al. [53]. We computed randomized quantile residuals with the R-package DHARMa [54], and inspected their distribution within and among factor predictor levels that are included or not in the models, and performed posterior predictive checks to assess model dispersion and overall model fit. Models were initially implemented using the most appropriate family distribution based on the nature of the response variable. The family was sometimes adjusted after model assessment to better capture the observed data.

Data from the first experiment originated from 42 individuals (24 *S. maderensis* and 18 *S. porcus*) that were used to test objectives 1 and 2 (see Introduction). Observations at the two time points (minute 1 and 5) were averaged since there was little variation between these two observations (Additional file 1: Figure S5). To assess changes in scorpionfish body luminance and hue (1), we implemented a generalised linear mixed model using a Gamma distribution (link = log) for the response variable *luminance*, and one using a Gaussian distribution for *hue.* Both models included the fixed effects *background (dark/grey, medium/orange, light/grey*), *scorpionfish species* (*S. maderensis*, *S. porcus*) and *observer* (*T. delaisi, P. flavescens*), and their interaction. *Fish ID* was used as a random intercept to account for the repeated measurements of each fish [55]. We further included a random slope over *background* in the *luminance* model, to account for differences in the predictor-response relationship between individual fish [56]. To assess how well scorpionfish matched their backgrounds (2), we implemented a generalised linear mixed model using a Gaussian distribution for the response variable *achromatic contrast,* and one using a Tweedie distribution (link = log) for *chromatic contrast.* The fixed effects and random intercept were identical as described above. We further included a random slope over *background* in the *chromatic contrast* model. For each model, random slopes were added when the differences in group means of interest varied among the random predictors’ levels.

We did not obtain fluorescence photos for 5 of the 42 individuals because of temporary technical difficulties with the photo-box and therefore used data from only 37 individuals (21 *S. maderensis* and 16 *S. porcus*) to test objective 3 (see Introduction) and assess changes in the response variable *fluorescent area* (i.e. the area of scorpionfish body showing fluorescence). For this model, we used a negative binomial distribution (link = log). Since this variable was not based on visual modelling, we here only included the fixed effects *background*, *species*, and their interaction. *Fish ID* was also included as random intercept.

We report R^2^-values as a measure of fit for each model and report both the marginal R^2^ (variance explained by fixed effects only) and the conditional R^2^ (variance explained by entire model) [57] (Table 1, 2 and Additional file 1: S1), using the *r2* function of the performance package [58]. For graphical displays of the results, our figures present model predicted means and their 95% compatibility (i.e. credible) intervals calculated from the posterior distributions of fitted values obtained from 10,000 sets of model parameters [52]. The same posterior distribution of fitted values was used to compute and report median differences between factor levels and their 95% compatibility intervals for all combinations of factor predictors of interest (Tables 1,2 and Additional file 1: S1). Effect size strength increases with increasing deviation of differences from zero, and the robustness of the result increases with decreasing degree of overlap of the 95% compatibility intervals (CIs) with zero. We refrain from reporting associated *p*-values because they offer limited information about the biological relevance of the observed effects [59, 60].

#### Experiment 2

We visualised data from 9 *S. maderensis* and 14 *S. porcus* to evaluate how fast scorpionfish adjust body luminance to the background (objective 4, see Introduction). One *S. porcus* was excluded from the graphs since it showed little change of luminance within one minute and did not seem to adjust to the background (absolute difference between t_0_ and t_60_ < 0.001 luminance channel cone catches). We calculated the proportional change in luminance at each time point (second 5, 10, 15, 20, 25), scaled for the total luminance change of every individual fish from initial to final luminance. We used the luminance channel cone catches of second 0 (t_0_) as the initial value for luminance and of second 60 (t_60_) as the final value for luminance, and calculated proportional change at time *t*_*x*_ as follows: proportional change *t*_*x*_ = (luminance *t*_*x *_− luminance *t*_*0*_)/(luminance *t*_*60 *_− luminance *t*_*0*_). We then plotted the medians and interquartile range of these proportional change values over time to display how much time was needed to complete a certain percentage of the overall achieved luminance change.

## Supplementary Information

**Additional file 1**. **Table S1**: Median differences in achromatic and chromatic contrasts. **Figure S2**: Reflectance spectra of the three backgrounds and the acclimation box. **Figure S3**: Photo-chamber for fluorescence photos. Supplementary Methods: ROI selection; **Figure S4**: Example of ROI selection. **Figure S5**: Luminance of scorpionfish body between time points.

## Data Availability

The datasets generated and analysed during the current study are available on Figshare: https://doi.org/10.6084/m9.figshare.22059092. The script used for statistical analysis can be found in Santon et al. [53].

## References

[1] Merilaita S, Stevens M, Stevens M, Merilaita S (2011). Crypsis through background matching. Anim camoufl mech funct.

[2] Stevens M, Merilaita S (2009). Animal camouflage: current issues and new perspectives. Philos Trans R Soc B Biol Sci.

[3] Duarte RC, Flores AAV, Stevens M (2017). Camouflage through colour change: mechanisms, adaptive value and ecological significance. Philos Trans R Soc B Biol Sci.

[4] Hughes A, Liggins E, Stevens M (2019). Imperfect camouflage: How to hide in a variable world?. Proc R Soc B Biol Sci.

[5] Nilsson Sköld H, Aspengren S, Cheney KL, Wallin M. Fish chromatophores—from molecular motors to animal behavior. In: Jeon KW (Eds.). Int Rev Cell Mol Biol. Chennai: Academic Press; 2016. p. 171–220. DOI: 10.1016/S1937-6448(08)01606-7 10.1016/bs.ircmb.2015.09.005 26811288

[6] Harant UK, Michiels NK, Anthes N, Meadows MG (2016). The consistent difference in red fluorescence in fishes across a 15 m depth gradient is triggered by ambient brightness, not by ambient spectrum. BMC Res Notes.

[7] Wuthrich KL, Nagel A, Swierk L (2022). Rapid body color change provides lizards with facultative crypsis in the eyes of their avian predators. Am Nat.

[8] Stuart-Fox D, Moussalli A, Whiting MJ (2008). Predator-specific camouflage in chameleons. Biol Lett.

[9] Chiao CC, Wickiser JK, Allen JJ, Genter B, Hanlon RT (2011). Hyperspectral imaging of cuttlefish camouflage indicates good color match in the eyes of fish predators. Proc Natl Acad Sci USA.

[10] Akkaynak D, Allen JJ, Mäthger LM, Chiao CC, Hanlon RT (2013). Quantification of cuttlefish (*Sepia officinalis*) camouflage: a study of color and luminance using in situ spectrometry. J Comp Physiol A Neuroethol Sens Neural Behav Physiol.

[11] Ramachandran VS, Tyler CW, Gregory RL, Duensing S, Pillsbury C, Ramachandran C (1996). Rapid adaptive camouflage in tropical flounders. Nature.

[12] Tyrie EK, Hanlon RT, Siemann LA, Uyarra MC (2015). Coral reef flounders, *Bothus lunatus*, choose substrates on which they can achieve camouflage with their limited body pattern repertoire. Biol J Linn Soc.

[13] Stevens M, Lown AE, Denton AM (2014). Rockpool gobies change colour for camouflage. PLoS ONE.

[14] Smithers SP, Rooney R, Wilson A, Stevens M (2018). Rock pool fish use a combination of colour change and substrate choice to improve camouflage. Anim Behav.

[15] Smithers SP, Wilson A, Stevens M (2017). Rock pool gobies change their body pattern in response to background features. Biol J Linn Soc.

[16] Anthes N, Theobald J, Gerlach T, Meadows MG, Michiels NK (2016). Diversity and ecological correlates of red fluorescence in marine fishes. Front Ecol Evol.

[17] Michiels NK, Anthes N, Hart NS, Herler J, Meixner AJ, Schleifenbaum F (2008). Red fluorescence in reef fish: A novel signalling mechanism?. BMC Ecol.

[18] Johnsen S (2012). The optics of life.

[19] Zawada DG, Mazel CH (2014). Fluorescence-based classification of caribbean coral reef organisms and substrates. PLoS ONE.

[20] Sparks JS, Schelly RC, Smith WL, Davis MP, Tchernov D, Pieribone VA (2014). The covert world of fish biofluorescence: a phylogenetically widespread and phenotypically variable phenomenon. PLoS ONE.

[21] Pembury Smith MQR, Ruxton GD (2020). Camouflage in predators. Biol Rev.

[22] Santon M, Bitton PP, Harant UK, Michiels NK (2018). Daytime eyeshine contributes to pupil camouflage in a cryptobenthic marine fish. Sci Rep.

[23] Marshall NJ, Carleton KL, Cronin TW (2015). Colour vision in marine organisms. Curr Opin Neurobiol.

[24] Schweikert LE, Fitak RR, Caves EM, Sutton TT, Johnsen S (2018). Spectral sensitivity in ray-finned fishes: diversity, ecology and shared descent. J Exp Biol.

[25] Louisy P (2002). Meeresfische Westeuropa und Mittelmeer.

[26] Neumann V, Paulus T. (2005) Mittelmeer Atlas Fische und ihre Lebensräume. In: Baensch HA (Eds), Melle, Germany: Mergus Verlag GmbH; 2005.

[27] Caves EM, Nowicki S, Johnsen S (2019). Von Uexküll revisited: addressing human biases in the study of animal perception. Integr Comp Biol.

[28] Nilsson Sköld H, Aspengren S, Wallin M (2013). Rapid color change in fish and amphibians—function, regulation, and emerging applications. Pigment Cell Melanoma Res.

[29] Wucherer MF, Michiels NK (2014). Regulation of red fluorescent light emission in a cryptic marine fish. Front Zool.

[30] Robledo-Ospina LE, Escobar-Sarria F, Troscianko J, Rao D (2017). Two ways to hide: predator and prey perspectives of disruptive coloration and background matching in jumping spiders. Biol J Linn Soc.

[31] Stevens M, Merilaita S (2009). Defining disruptive coloration and distinguishing its functions. Philos Trans R Soc B Biol Sci.

[32] Cott HB (1940). Adaptive coloration in animals.

[33] Govardovskii VI, Zueva LV (1988). Photoreceptors and visual pigments in fish from the black-sea. J Evol Biochem Physiol.

[34] Whiteley AR, Gende SM, Gharrett AJ, Tallmon DA (2009). Background matching and color-change plasticity in colonizing freshwater sculpin populations following rapid deglaciation. Evolution.

[35] da Silva CRB, van den Berg CP, Condon ND, Riginos C, Wilson RS, Cheney KL (2020). Intertidal gobies acclimate rate of luminance change for background matching with shifts in seasonal temperature. J Anim Ecol.

[36] Siddiqi A, Cronin TW, Loew ER, Vorobyev M, Summers K (2004). Interspecific and intraspecific views of color signals in the strawberry poison frog *Dendrobates pumilio*. J Exp Biol.

[37] Vorobyev M, Osorio DC (1998). Receptor noise as a determinant of colour thresholds. Proc R Soc B Biol Sci.

[38] Maia R, Gruson H, Endler JA, White TE (2019). pavo 2: new tools for the spectral and spatial analysis of colour in R. Methods Ecol Evol.

[39] R Core Team. R: A language and environment for statistical computing.R Foundation for Statistical Computing, Vienna, Austria. URL https://www.r-project.org/. 2021.

[40] Lyall AH (1957). Cone arrangements in teleost retinae. J Cell Sci.

[41] Troscianko J, Stevens M (2015). Image calibration and analysis toolbox—a free software suite for objectively measuring reflectance, colour and pattern. Methods Ecol Evol.

[42] Schneider CA, Rasband WS, Eliceiri KW (2012). NIH Image to ImageJ: 25 years of image analysis. Nat Methods.

[43] Bitton PP, Harant UK, Fritsch R, Champ CM, Temple SE, Michiels NK (2017). Red fluorescence of the triplefin *Tripterygion delaisi* is increasingly visible against background light with increasing depth. R Soc Open Sci..

[44] Utne-Palm AC, Bowmaker JK (2006). Spectral sensitivity of the two-spotted goby *Gobiusculus flavescens* (Fabricius): a physiological and behavioural study. J Exp Biol.

[45] Champ CM, Vorobyev M, Marshall NJ (2016). Colour thresholds in a coral reef fish. R Soc Open Sci..

[46] Olsson P, Lind O, Kelber A (2018). Chromatic and achromatic vision: Parameter choice and limitations for reliable model predictions. Behav Ecol.

[47] Fritsch R, Collin SP, Michiels NK (2017). Anatomical analysis of the retinal specializations to a crypto-benthic, micro-predatory lifestyle in the Mediterranean triplefin blenny *Tripterygion delaisi*. Front Neuroanat.

[48] Green SD, Duarte RC, Kellett E, Alagaratnam N, Stevens M (2019). Colour change and behavioural choice facilitate chameleon prawn camouflage against different seaweed backgrounds. Commun Biol.

[49] Lythgoe JN (1979). The ecology of vision.

[50] Pierotti MER, Wandycz A, Wandycz P, Rebelein A, Corredor VH, Tashiro JH (2020). Aggressive mimicry in a coral reef fish: the prey’s view. Ecol Evol.

[51] van den Berg CP, Troscianko J, Endler JA, Marshall NJ, Cheney KL (2020). Quantitative colour pattern analysis (QCPA): a comprehensive framework for the analysis of colour patterns in nature. Methods Ecol Evol.

[52] Brooks M, Kristensen K, van Benthem K, Magnusson A, Berg C, Nielsen A (2017). glmmTMB balances speed and flexibility among packages for zero-inflated generalized linear mixed modeling. R J.

[53] Santon M, Korner-Nievergelt F, Michiels NK, Anthes N. A versatile workflow for linear modelling in R. Front Ecol Evol. 2023;11:1065273. 10.3389/fevo.2023.1065273.

[54] Hartig F. DHARMa: residual diagnostics for hierarchical (Multi-Level/Mixed) regression models. R package version 0.4.5. http://florianhartig.github.io/DHARMa/. 2022

[55] Schielzeth H, Forstmeier W (2009). Conclusions beyond support: overconfident estimates in mixed models. Behav Ecol.

[56] Korner-Nievergelt F, Roth T, von Felten S, Guélat J, Almasi B, Korner-Nievergelt P. Bayesian data analysis in ecology using linear models with R, BUGS, and Stan. Academic Press; 2015.

[57] Nakagawa S, Schielzeth H (2013). A general and simple method for obtaining R2 from generalized linear mixed-effects models. Methods Ecol Evol.

[58] Lüdecke D, Ben-Shachar M, Patil I, Waggoner P, Makowski D (2021). Performance: an R package for assessment, comparison and testing of statistical models. J Open Source Softw.

[59] Halsey LG (2019). The reign of the p-value is over: What alternative analyses could we employ to fill the power vacuum?. Biol Lett.

[60] Berner D, Amrhein V (2022). Why and how we should join the shift from significance testing to estimation. J Evol Biol.

