# Supplementary material for: Scorpionfish rapidly change colour in response to their background
Source: Front Zool. 2023 Mar 3;20:10. doi: 10.1186/s12983-023-00488-x (PMC9983180; doi:10.1186/s12983-023-00488-x)
Supplement: Supplementary file 1 — Additional file 1. Table S1: Median differences in achromatic and chromatic contrasts. Figure S2: Reflectance spectra of the three backgrounds and the acclimation box. Figure S3: Photo-chamber for fluorescence photos. Supplementary Methods: ROI selection; Figure S4: Example of ROI selection. Figure S5: Luminance of scorpionfish body between time points. [file 12983_2023_488_MOESM1_ESM.pdf]

Scorpionfish rapidly change colour in response to their background

Additional file 1: Supplementary material

Table S1: Median differences in achromatic and chromatic contrasts of scorpionfish body against all backgrounds.

	Scorpaena maderensis			***Scorpaena porcus***		
Background	Median	Lower CIs	Upper CIs	Median	Lower CIs	Upper CIs
**A) Achromatic contrast.** R^2_{marg} = 0.659, R^2_{cond} = 0.969						
Observer = T. delaisi						
medium/orange - dark/grey	-15.98	-19.53	-12.45	-8.61	-12.66	-4.53
medium/orange - light/grey	-14.92	-16.26	-13.54	-16.78	-18.35	-15.24
light/grey - dark/grey	-1.08	-5.12	3.04	8.17	3.47	12.91
Observer = P. flavescens						
medium/orange - dark/grey	-16.14	-19.75	-12.66	-8.46	-12.62	-4.45
medium/orange - light/grey	-12.90	-14.25	-11.51	-14.07	-15.58	-12.49
light/grey - dark/grey	-3.29	-7.31	0.78	5.66	0.95	10.19
**B) Chromatic contrast.** R^2_{marg} = 0.873, R^2_{cond} = 0.981						
Observer = T. delaisi						
medium/orange - dark/grey	0.52	0.24	0.79	1.19	0.89	1.51
medium/orange - light/grey	0.85	0.55	1.15	1.27	0.93	1.62
light/grey - dark/grey	-0.33	-0.47	-0.20	-0.07	-0.21	0.07
Observer = P. flavescens						
medium/orange - dark/grey	-0.58	-1.31	0.14	1.47	0.70	2.30
medium/orange - light/grey	2.03	1.37	2.69	3.37	2.62	4.17
light/grey - dark/grey	-2.61	-2.97	-2.28	-1.90	-2.23	-1.57

Median differences of A) achromatic contrast and B) chromatic contrast between all combinations of *background*, *species* and *observer*. Estimated effect sizes are reported as the median difference and its 95% compatibility intervals (CIs), calculated from 10,000 simulations of the posterior distribution of model parameters. N = 24 for *S. maderensis* and N = 18 for *S. porcus*. Effect size strength increases with increasing deviation of median differences from zero, and the robustness of the result increases with decreasing degree of overlap of the 95% compatibility intervals (CIs) with zero.

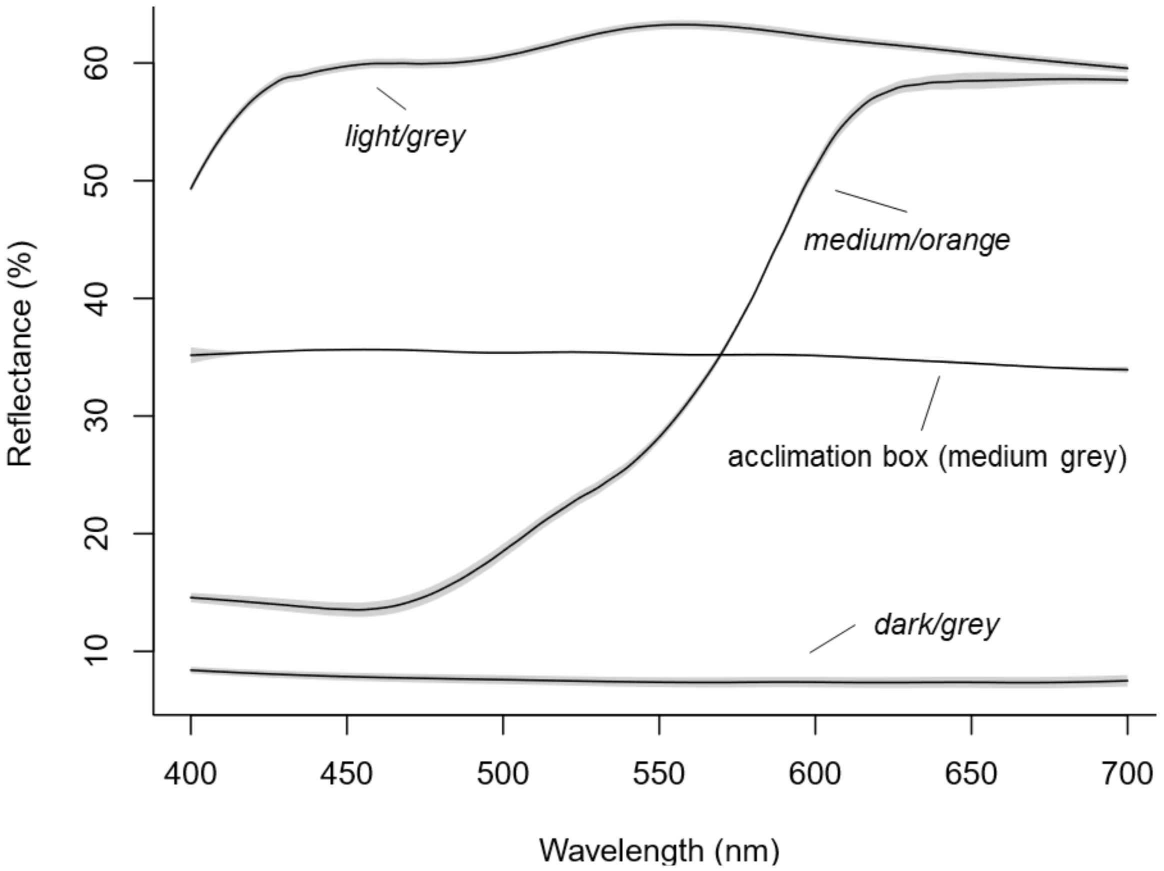

Figure S2: Reflectance spectra of the three backgrounds and the acclimation box. The *medium/orange* background has intermediate luminance and is the only one showing a strong chromatic component. Shown are the mean spectra (black lines) and standard deviation (grey) from three measurements per background and box.

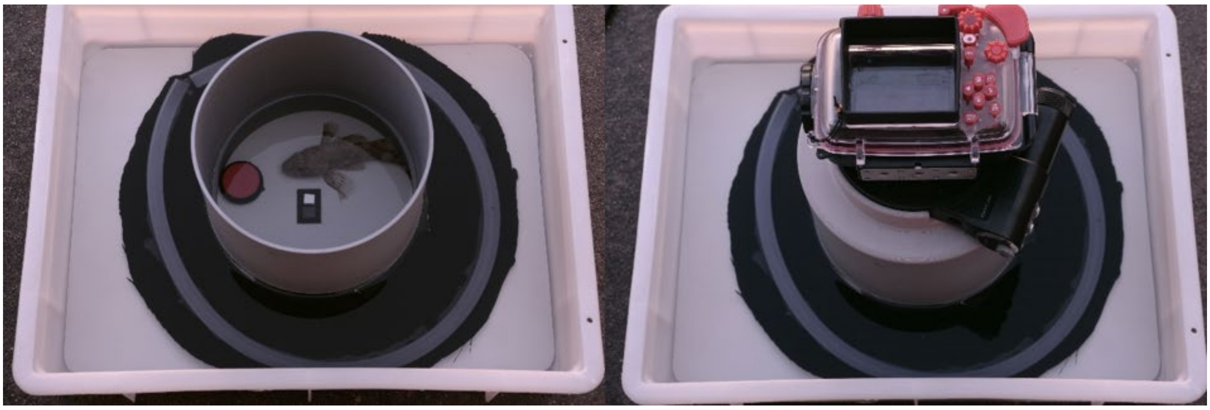

Figure S3: Photo-chamber for fluorescence photos. The cylindrical chamber was placed over the fish and the red standard placed next to the fish (left), before the chamber was closed with the lid equipped with the light source and camera (right).

Supplementary Methods: ROI selection

To allow reliable image analysis, the analysed ROIs were chosen in the same plane as the grey standards used to normalise the images. The location of the ROI ‘background’ was chosen to be close to the fish but not shaded by it, and always selected in the corner that was closest to the centre of the tray (Figure S4). Since fishes have a 3D body shape, we had to be cautious when selecting the ‘body’ area that was meant to be analysed. Before taking a photo, we assured that fish would sit in the centre of the tray by gently guiding the fish using a stick, if needed. With the fish sitting upright at the centre of the tray, and with the top-down view of the camera, we ensured that the fish body was approximately in the same plane as the grey standards in the images. While selecting the area, we ensured that no marginal part of the fish, which could have been influenced by the background, was included (Figure S4).

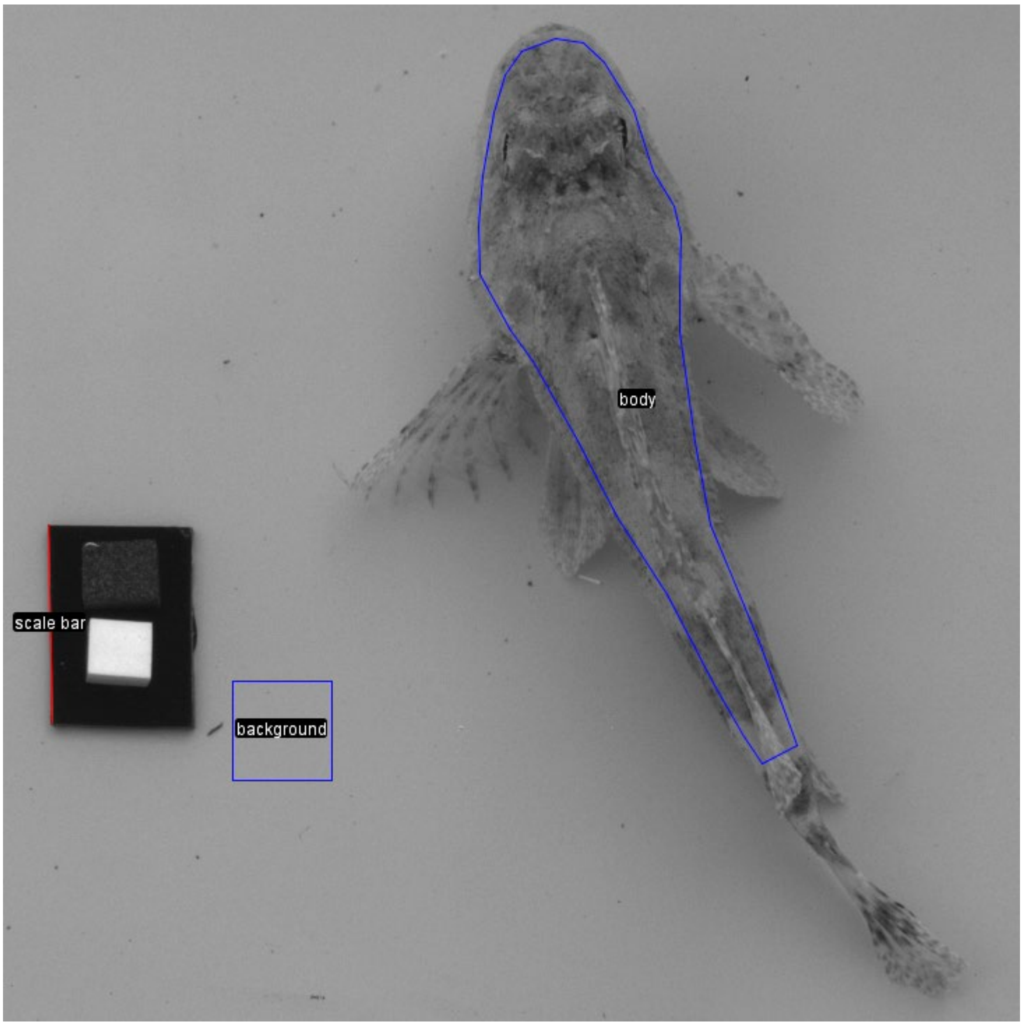

Figure S4: Example of ROI selection in a *Scorpaena maderensis* individual on the *light/grey* background. Indicated are the scale bar (red line, 2 cm) next to the grey standards, the ROIs ‘background’ and ‘body’.

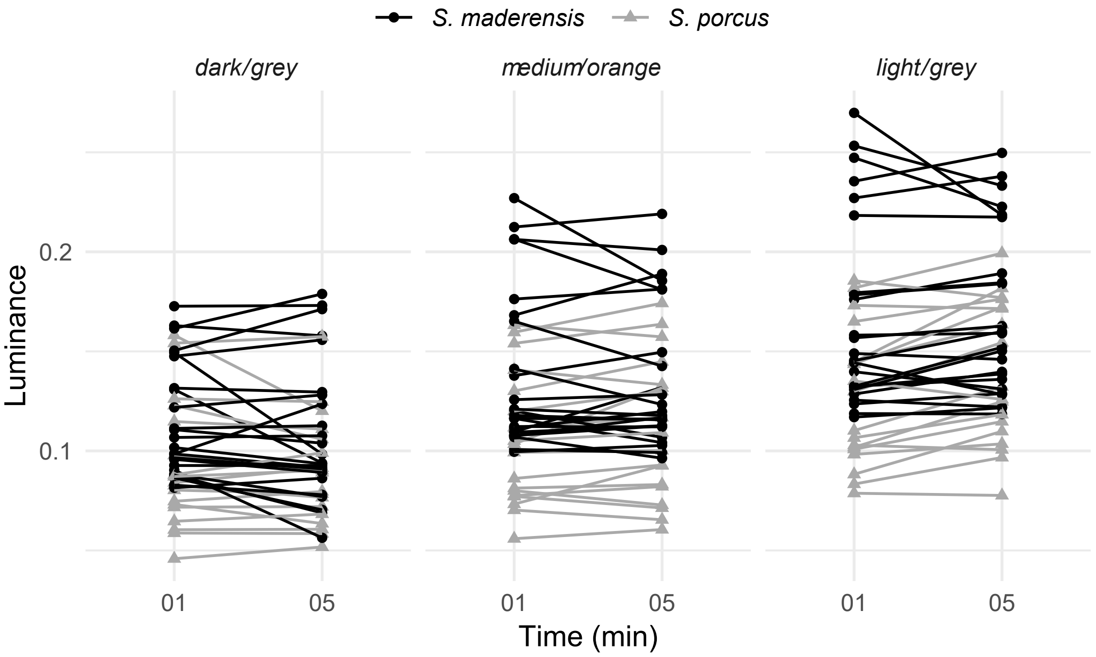

Figure S5: Luminance of scorpionfish body does not differ between one and five minutes on each background. Points (*Scorpaena maderensis*) and triangles (*S. porcus*) represent individual luminance channel cone catches of a scorpionfish for *Tripterygion delaisi* vision. Measurements of an individual on the same background at the two time points are connected with a line.
